# Short-Term Benefits of Continuous Positive Airway Pressure Treatment on Cognition in the Obstructive Sleep Apnea Syndrome: A Retrospective Study

**DOI:** 10.3390/brainsci13010124

**Published:** 2023-01-10

**Authors:** Giulia Vaioli, Sofia Tagini, Federica Scarpina, Riccardo Cremascoli, Lorenzo Priano, Mauro Cornacchia, Paolo Fanari, Alessandro Mauro

**Affiliations:** 1 I.R.C.C.S. Istituto Auxologico Italiano, U.O. di Neurologia e Neuroriabilitazione, Ospedale San Giuseppe, 28824 Piancavallo, VCO, Italy; 2 “Rita Levi Montalcini” Department of Neurosciences, University of Turin, 10124 Turin, Italy; 3 I.R.C.C.S. Istituto Auxologico Italiano, U.O. di Riabilitazione Pneumologica, Ospedale S. Giuseppe, 28824 Piancavallo, VCO, Italy

**Keywords:** ventilotherapy, continuous positive airway pressure, obstructive sleep apnea syndrome, cognitive deficits

## Abstract

The Obstructive Sleep Apnea Syndrome (OSAS) significantly impacts cognitive functioning. The prolonged use (more than 3 months) of ventilotherapy with continuous positive airway pressure (CPAP) seems to have positive effects in restoring cognitive difficulties. However, there is poor evidence about its possible short-term effect. We investigated whether the short use (less than 15 days at testing) of CPAP improved the cognitive functioning in fifty individuals with OSAS by collecting retrospective neuropsychological measures about verbal memory and learning, information processing speed, attention (i.e., alerting, orienting, and executive system), and executive functions (i.e., strategic reasoning, problem-solving, and mental planning). The predictive role of days of CPAP use on the neuropsychological scores was assessed by hierarchical multiple linear regression analyses, over and above the possible role of demographics, body mass index, level of OSAS severity, and the level of anxiety and depression. The average number of days since CPAP adaptation was 4.70 (SD = 3.90; range = 0–15). As the days of CPAP adaptation increased, verbal learning and long-term memory significantly improved, contrary to the other assessed domains. Our results show a significant improvement in some cognitive functions even after a short treatment with CPAP, pointing to the importance of the early use of ventilotherapy to rapidly improve cognitive functioning. Identifying which cognitive functions can or cannot be restored with CPAP use may enable the design of complementary neuropsychological interventions focused on those residual difficulties, possibly enhancing patients’ compliance to the treatment.

## 1. Introduction

Ventilotherapy with continuous positive airway pressure (CPAP) represents the most effective treatment in the case of Obstructive Sleep Apnea Syndrome (OSAS), regardless of comorbidities and the severity of respiratory symptoms [1]. Crucially, this treatment improves not only the respiratory symptoms, but it seems to restore those cognitive difficulties generally associated with OSAS [2,3,4,5,6,7].

The improvement of cognitive impairments after long-term CPAP treatment (i.e., three-to-six months) has been widely documented [2,3,4,7,8,9,10,11,12]. On the contrary, the evidence about the short-term benefits (i.e., after less than two weeks of treatment) of CPAP on the cognitive difficulties in OSAS is more limited and quite dated. A short CPAP intervention (<15 days) seems especially related to improvement in vigilance (i.e., day-time sleep latency, as measured by the Multiple Sleep Latency Test [13]) [4,6] and sustained attention (i.e., the ability to maintain attention while performing a specific task) [3,14,15]. However, short-term and long-term memory and executive functions seem less sensitive to brief treatments [14,16]. Concerning psychomotor processing and visuospatial abilities, the evidence about a quick effect of CPAP treatment is controversial [3,14,16].

To sum up, the previous evidence suggests that CPAP may have a short-term beneficial effect on certain cognitive abilities, whereas some other cognitive domains seemed not to improve. From a neuropsychological perspective, identifying the cognitive functions that can be shortly improved with the CPAP treatment and those that are not susceptible to a quick reversibility may enable the design of more efficient neuropsychological rehabilitative interventions, focused on those cognitive difficulties that cannot be improved with the CPAP use alone. This is especially valuable considering that neuropsychological rehabilitative treatments are usually effortful and time-consuming. From a clinical perspective, enhancing the short-term cognitive benefits of the CPAP therapy through informed communications may improve patients’ compliance. Many patients complain about the discomfort of the device, and adherence to the CPAP treatment is typically low [1] (i.e., 8 to 15% of patients refuse CPAP treatment after the first usage and at least 50% of them do not use the device regularly within 1 year of treatment [17]). Acknowledging the possible, upcoming advantages of the CPAP treatment on daily cognitive functioning, and thus quality of life, may facilitate the process of compliance to the treatment. Thus, we suggest that a more comprehensive and updated understanding of the possible short-term effect of CPAP treatment on the patients’ cognitive functioning is very relevant.

The aim of this research was to provide further and updated evidence about the possible association between a short-term (i.e., zero to 15 days) CPAP treatment in individuals affected by OSAS and cognitive functioning. To this purpose, we collected retrospective data concerning the cognitive profile of individuals with OSAS, as previously assessed with standard neuropsychological tests for other research purposes, who were about to start or had recently begun the CPAP therapy. The following cognitive domains were considered: verbal memory and learning; information processing speed; sustained, selective, and attentional executive control; and the executive functions of strategic reasoning, problem-solving, and mental planning. According to several pieces of evidence, these cognitive domains are severely affected by OSAS [18,19,20,21,22,23,24] and they significantly impact individuals’ daily functioning and, possibly, the management of the CPAP. Additionally, in this research, the patients’ self-reported evaluation of psychological distress (i.e., depressive symptoms and anxiety) was recorded, given its possible confounding effect on cognitive functioning.

## 2. Materials and Methods

### 2.1. Participants

Participants with OSAS included in this retrospective study had been recruited between April 2021 and March 2022 at the I.R.C.C.S. Istituto Auxologico Italiano, Ospedale San Giuseppe (Piancavallo, VB, Italy) to be involved in a previous research protocol of our group. Inpatients were recruited consecutively, but only those satisfying the inclusion/exclusion criteria were enrolled and tested at the beginning of their rehabilitative recovery. In line with previous investigations by our research group [25,26], only patients with an overall cognitive functioning within the normal range (i.e., MoCA score > 15.5 [27]) and presenting more than 5 apnea and hypopnea events per hour of sleep during a full-night polysomnography (i.e., the apnea-hypopnea index—AHI [28]) were included. AHI values between 5 ≤ AHI < 15 and 15 ≤ AHI < 30 define mild and moderate apnea, respectively; AHI values ≥ 30 define severe apneas [28]. Individuals who used ventilotherapy in the past and who declared a regular use of hypnotic medications were excluded. As well, smokers; individuals with a history of alcohol abuse; and individuals with a history of cardiovascular, psychiatric, neurological disorders, or any concurrent medical condition that was not related to OSAS were excluded.

As part of their rehabilitative protocol, all participants were about to start or had been using the CPAP device for a few days. The process of adaptation to the use of the device has been previously described in detail [26]. Briefly, trained nurses illustrated the CPAP component and functioning to the patients and assisted them in wearing the mask comfortably and autonomously using the CPAP, enhancing the benefits associated with the treatment. During the night, the nursing staff supervised patients in ventilotherapy and verified whether the CPAP had been used correctly through the CPAP output.

For the aim of this retrospective study, we computed the number of days between the CPAP first use and the neuropsychological testing for each participant, according to patients’ clinical records. The time of usage in hours would be more informative since the actual use of the device during the night might differ across participants; however, patients’ clinical records only notified when the treatment began. The neuropsychological assessment was conducted as part of a different research protocol: each participant underwent the examination at different timings relative to the beginning of the CPAP treatment (see Table 1). All participants were tested before or during the first two weeks of CPAP therapy.

### 2.2. Demographic and Clinical Measures

Basic demographic information (i.e., sex, age, and years of education) were available and included in the study considering their possible interplay with cognition [29,30]. Additionally, the body mass index (i.e., kg/m^2^—BMI) was collected, which is informative of the presence of obesity. Indeed, this is a clinical condition frequently associated with both OSAS [31] and cognitive impairments [32,33].

The AHI and the cumulative time with a peripheral blood oxygen saturation (SpO_2_) below 90% while sleeping, as measured at the admission, were considered indices of sleep apnea severity [34,35].

### 2.3. Neuropsychological Measures

Performance scores on neuropsychological tests administered by a trained neuropsychologist were collected. The neuropsychological tests were presented in a random order, to overcome the bias related to the order of administration. Eight different blocks have been identified and repeated across participants. Between the immediate recall and the deferred recall of the verbal memory test, non-linguistic tasks were always performed to avoid any interference with verbal learning. Overall, participants took an hour to complete the entire protocol.

The Selective Reminding Test (SRT [36]) measures verbal memory and learning using a list-learning procedure across successive trials. Three indices are provided: (*i*) the long-term storage capacity (SRT-LTS) as a measure of encoding efficiency, (*ii*) the consistency of long-term retrieval in successive trials (SRT-CLTR) as a measure of verbal learning ability, and (*iii*) the delay recall of information (SRT-D) as a measure of the persistence of learning in time. Higher scores are indicative of more efficient verbal memory and learning processes.

The Symbol Digit Modality Test (SDMT [37]) measures the information processing speed in the visuo-spatial domain. Higher scores indicate faster information processing.

The Attention Network Test (ANT [38]) is a computer-based assessment designed to evaluate the efficiency of three independent attentional networks within a single 30 min testing session: (*i)* the alerting (ANT-Al) defined as reaching and maintaining a state of alert (i.e., sustained attention); (*ii)* orienting (ANT-Or), the ability to select information from sensory inputs (i.e., selective attention); (*iii)* the executive (ANT-Ex) system, defined as resolving the conflict between responses (i.e., attentional executive control). Reaction times are used to quantify the processing efficiency within each attentional network: lower scores suggest a faster reaction time, thus more efficient attentional processing.

The Tower of London (ToL [39]) assesses strategic reasoning, problem-solving, and mental planning. Both the time (i.e., the speed; *ToL-Time*) and the number of attempts (i.e., the accuracy; *ToL-Acc*) necessary to find a solution are computed.

### 2.4. Psychological Measures

Since the psychological comorbidities often observed in the OSAS [40] possibly affect the cognitive performance [41,42], the self-reported level of anxiety and depressive symptoms were recorded at the end of the neuropsychological assessment.

The State-Trait Anxiety Inventory (STAI [43]) was used to assess both the state (20 items; STAI-Y1 form) and the trait (20 items; STAI-Y2 form) of anxiety. Items are rated on a four-point Likert-type scale according to the intensity of symptoms in the present moment (STAI-Y1, from “not at all” to “very much so”) or the overall frequency of symptoms in every-day life (*STAI-Y2*, from “almost never” to “almost always”); higher scores represent a higher level of anxiety. Only trait anxiety (STAI-Y2) was considered in the present work as we were interested in how anxiety may interplay with cognitive functions daily, rather than during the neuropsychological assessment. From a clinical perspective, we believe it may be more useful collecting data about the patient’s usual functioning since rehabilitative treatments should aim to improve individuals’ overall quality of life.

The presence of depressive symptoms in the past week was evaluated with the Beck Depression Inventory (BDI [44]): a self-report measure of cognitive and somatic depressive symptoms including twenty-one items. For each item, participants choose the option that better represents how he/she has been feeling in the past week (e.g., I do not feel sad/I feel sad/I am sad all the time and I can’t snap out of it/I am so sad and unhappy that I can’t stand it); higher scores represent a higher level of depressive symptoms.

### 2.5. Analyses

The possible role of days of CPAP ventilotherapy in predicting participants’ performance in selected cognitive functions was identified through a series of hierarchical multiple linear regression analyses.

In this analytical approach, independent variables (i.e., the predictors) are entered in the regression model sequentially, allowing the identification of the unique contribution of each predictor (or set of predictors) on the dependent variable at each step (i.e., block), over and above those considered in the previous steps. Analyses of regression were performed for each cognitive domain independently: the group’s average score in each cognitive test or sub-test of interest was assumed as a dependent variable. In each analysis, in the first block, demographic (i.e., Sex, Age, Education, and BMI) and clinical variables informative of OSAS severity (i.e., AHI, % Time with SpO_2_ < 90%) were considered as possible predictors (Model 1). In block 2, the possible contribution of psychological aspects on cognitive performance was introduced by entering as additional predictors the level of trait anxiety (i.e., *STAI-Y2* score) and depressive symptoms (i.e., BDI score) (Model 2). Finally, in block 3, we considered the possible contribution of days since CPAP as the first adaptation on cognitive functioning, over and above demographic, clinical, and psychological variables (Model 3).

Multicollinearity was checked by inspecting VIF values for each independent variable; a VIF value higher than 10 was considered informative of possible multicollinearity [45]. Linearity was assessed by a visual inspection of partial regression plots and by plotting and inspecting studentized residuals against the unstandardized predicted values; homoscedasticity was assessed by plotting and inspecting studentized residuals versus unstandardized predicted values. Univariate outliers were identified by studentized residuals greater than ±3. Relevant leverage values (centered leverage value > 0.2 [46]) and highly influential points (i.e., Cook’s distance above 1 [47]) were recorded. The normality of residuals was assessed by a visual inspection of the Q-Q plot.

The partial correlation coefficient *r* for each independent variable and the adjusted percentage of explained variance (R^2^) for each block were reported as a measure of effect size; effect sizes were interpreted according to the guidelines [48].

## 3. Results

Fifty participants with OSAS were included in the study (age: M = 58.62, SD = 10.95; years of education: M = 11.30, SD = 4.06; BMI: M = 45.41, SD = 7.74; AHI: M = 54.46, SD = 30.95; % Time SpO_2_ < 90%: M = 53.26, SD = 29.81), and of them, twenty-three were female. The AHI was considered informative of apneas severity according to standard criteria (ref): most of the sample had severe apnea (*n* = 35, 70%), whereas a minority had moderate (*n* = 11, 22%) or mild (*n* = 4, 8%) apnea. Days since the first CPAP adaptation ranged between 0 and 15 days (M = 4.70, SD = 3.90). Means, standard deviations (SD), and range values of the psychological and neuropsychological tests’ scores for the whole sample (*n* = 50) are reported in Table 2.

### 3.1. Preliminary Analysis

Overall, there was no evidence of multicollinearity (i.e., all VIF values were <10 [45]). Assumptions of linearity, homoscedasticity, and normality were met, unless otherwise specified in each regression model; in case the assumptions were not met, the transformations adopted have been reported. If outliers were detected (i.e., studentized residuals > ±3), details on how they were treated in each regression model have been specified; if not, no outliers were detected. Six observations were associated with high leverage (centered leverage value > 0.2 [46]); however, these cases were always included in the analyses since in none of the regression models these observations were highly influential (i.e., Cook’s distance < 1 [47]).

### 3.2. Verbal Memory and Learning

The full model (Model 3) investigating the contribution of days since the first CPAP adaptation in predicting the long-term memory storage capacity (i.e., SRT-LTS scores), over and above demographic (i.e., Sex, Age, Education, BMI), clinical (AHI, % Time with SpO_2_ < 90%), and psychological (STAI-Y2 and BDI scores) variables was statistically significant (R^2^ = 0.47, F (9,40) = 3.98, *p* = 0.001, adjusted R^2^ = 0.35). As shown in Table 3, *Age* (β = −0.48, *t* = 3.23, *p* = 0.002, partial *r* = −0.45) was a significant predictor of SRT-LTS scores with medium effect size, suggesting that as age increases, the long-term storage capacity decreases. In addition, Days of Adaptation (β = 0.31, *t* = 2.14, *p* = 0.039, partial *r* = 0.32) were a significant predictor of SRT-LTS scores with a medium effect size, suggesting that as the time using the CPAP increases, the long-term storage capacity improves.

Concerning verbal learning, the full model (Model 3) investigating the contribution of days since the first CPAP adaptation in predicting the SRT-CLTR scores, over and above demographic (i.e., Sex, Age, Education, BMI), clinical (AHI, % Time with SpO_2_ < 90%), and psychological (STAI-Y2 and BDI scores) variables was statistically significant (R^2^ = 0.54, F (9,40) = 5.2, *p* = 0.001, adjusted R^2^ = 0.47). As reported in Table 4, Age was a significant predictor of the SRT-CLTR score with a medium effect size (β = −0.67, *t* = 3.47, *p* = 0.001, partial *r* = −0.48), suggesting that as age increases the consistency of long-term retrieval across time decreases. Additionally, Days of Adaptation were a significant predictor of SRT-CLTR scores with a medium effect size (β = 0.27, *t* = 2.05, *p* = 0.047, partial *r* = 0.31), suggesting that as the time using the CPAP increases, the consistency of long-term retrieval across time (i.e., the verbal learning ability) improves.

Finally, the full model (Model 3) investigating the contribution of days since the first CPAP adaptation in predicting the persistence of learning in time (i.e., the SRT-D scores), over and above demographic (i.e., Sex, Age, Education, BMI), clinical (AHI, % Time with SpO_2_ < 90%), and psychological (STAI-Y2 and BDI scores) variables was statistically significant (R^2^ = 0.51, F (9,40) = 4.59, *p* = 0.001, adjusted R^2^ = 0.40). As shown in Table 5, Age was a significant predictor of the SRT-D scores with a medium effect size (β = −0.46, *t* = 3.23, *p* = 0.003, partial *r* = −0.45), suggesting that as age increases, the persistence of learning in time decreases. Furthermore, Days of Adaptation were a significant predictor of SRT-D scores with a medium effect size (β = 0.31, *t* = 2.24, *p* = 0.031, partial *r* = 0.33), suggesting that as the time using the CPAP increases, the persistence of learning in time improves.

### 3.3. Information Processing Speed

The full model (Model 3) investigating the contribution of days since the first CPAP adaptation in predicting the information processing speed (i.e., SDMT scores), over and above demographic (i.e., Sex, Age, Education, BMI), clinical (AHI, % Time with SpO_2_ < 90%), and psychological (STAI-Y2 and BDI scores) variables was statistically significant (R^2^ = 0.41, F (9,40) = 3.11, *p* = 0.006, adjusted R^2^ = 0.28). However, as shown in Table 6, only Age was a significant predictor of the SDMT scores with a medium effect size (β = −0.41, *t* = 2.54, *p* = 0.015, partial *r* = −0.37), suggesting that as age increases the information processing speed diminishes.

### 3.4. Attentional Networks

The full model (Model 3) investigating the contribution of days since the first CPAP adaptation in predicting the efficiency of the alerting attentional network (i.e., the ANT-Al scores), over and above demographic (i.e., Sex, Age, Education, BMI), clinical (AHI, % Time with SpO_2_ < 90%), and psychological (STAI-Y2 and BDI scores) variables was not statistically significant (R^2^ = 0.20, F (9,40) = 1.14, *p* = 0.36, adjusted R^2^ = 0.03), suggesting that none of the predictors included in the model significantly contribute to the ability to achieve and maintain alerts (see Table S1).

Concerning the ANT-Or scores, one outlier value was detected (i.e., standardized residual = 3.40), but since that case was not highly influential (i.e., Cook’s distance = 0.28) it was kept in the analysis. The full model (Model 3) investigating the contribution of days since the first CPAP adaptation in predicting the efficiency of the orienting attentional network (i.e., the ANT-Or scores), over and above demographic (i.e., Sex, Age, Education, BMI), clinical (AHI, % Time with SpO_2_ < 90%), and psychological (STAI-Y2 and BDI scores) variables was not statistically significant (R^2^ = 0.32, F (9,40) = 2.16, *p* = 0.051, adjusted R^2^ = 0.17), suggesting that none of the predictors included in the model significantly contribute to the ability to select information sources (see Table S2).

Finally, heteroscedasticity was detected by plotting and visually inspecting studentized residuals versus unstandardized predicted values for ANT-Ex scores; however, a logarithmic transformation [49] was applied and satisfactory homoscedasticity was achieved. The full model (Model 3) investigating the contribution of days since the first CPAP adaptation in predicting the efficiency of the executive attentional network (i.e., ANT-Ex scores), over and above demographic (i.e., Sex, Age, Education, BMI), clinical (AHI, % Time with SpO_2_ < 90%), and psychological (STAI-Y2 and BDI scores) variables was statistically significant (R^2^ = 0.39, F (9,40) = 2.81, *p* = 0.012, adjusted R^2^ = 0.25). However, as shown in Table 7, only Age (β = 0.51, *t* = 3.19, *p* = 0.003, partial *r* = −0.45) and BMI (β = 0.37, *t* = 2.42, *p* = 0.020, partial *r* = 0.36) were significant predictors of the ANT-Ex scores with a medium effect size, suggesting that as age and body mass index increase, the efficiency of executive control decreases (i.e., reaction time associated with executive control increases).

### 3.5. Strategical Reasoning, Problem-Solving, and Mental Planning

Heteroscedasticity was detected by plotting and visually inspecting studentized residuals versus unstandardized predicted values of the ToL-Time scores; however, the logarithmic transformation [49] was applied and satisfactory homoscedasticity was achieved. The full model (Model 3) investigating the contribution of days since the first CPAP adaptation in predicting the speed associated with strategical reasoning, problem-solving, and mental planning (i.e., ToL-Time scores), over and above demographic (i.e., Sex, Age, Education, BMI), clinical (AHI, % Time with SpO_2_ < 90%), and psychological (STAI-Y2 and BDI scores) variables was not statistically significant (R^2^ = 0.24, F (9,40) = 1.39, *p* = 0.226, adjusted R^2^ = 0.07) (see Table S3).

Concerning the accuracy of these processes, one univariate outlier value was detected (i.e., standardized residual = 3.24) for the ToL-Acc scores, but since that case was not highly influential (i.e., Cook’s distance = 0.15 [47]) it was kept in the analysis. The full model (Model 3) investigating the contribution of days since the first CPAP adaptation in predicting the ToL-Acc scores, over and above demographic (i.e., Sex, Age, Education, BMI), clinical (AHI, % time with SpO_2_ < 90%), and psychological (STAI-Y2 and BDI scores) variables was not statistically significant (R^2^ = 0.28, F (9,40) = 1.17, *p* = 0.119, adjusted R^2^ = 0.12) (see Table S4).

Taken together, our results suggest that age is the most frequent predictor of cognitive functions: as age increases, the verbal long-term memory storage capacity, consistency of retrieval (i.e., the verbal learning ability), persistence of verbal learning, information processing speed, and the efficiency of executive control diminish; furthermore, as BMI increases, the efficiency of executive control decreases. Concerning days of CPAP adaptation, we observed a specific positive association between the treatment and verbal learning and long-term memory, suggesting that cognition might benefit from short-term ventilotherapy, at least considering certain cognitive domains.

Post hoc power analyses relative to each of the regression models performed were computed with G*Power software (version 3.1 [50]). Considering 50 participants, 9 predictors, an alpha level of 0.05, and the f^2^ effect size estimated according to the observed R^2^, a satisfying power was reached relative to the three sub-scores of the SRT (99%), the SDMT (97%), the executive (93%), and the orienting (88%) indices of the ANT. Lower power was achieved for the regression models relative to the alerting score of the ANT (57%) and the three sub-scores of the ToL (57–78%).

## 4. Discussion

In this retrospective study, we provided more recent evidence about the possible association between short-term CPAP therapy and cognitive functioning in OSAS, through a clinical approach.

Our results showed that as the time using the CPAP increased, the participants’ verbal learning abilities and verbal long-term memory improved. Hence, ventilotherapy might improve patients’ cognitive performance even after a few days of usage. However, this short-term benefit might be limited to certain cognitive functions, such as verbal learning and long-term verbal memory (i.e., here measured by the Selective Reminding Test [36]). These results are consistent with previous studies reporting improving long-term verbal memory abilities after a prolonged CPAP treatment (3–6 months [2,8,12,51]), but not with those studies assessing the short-term benefit of ventilotherapy, which instead did not report any improvement in these cognitive domains [6,14]).

Nonetheless, the short CPAP therapy was not related to participants’ performance in the other cognitive domains investigated (i.e., information processing speed, sustained and selective attention, attentional executive control, and strategic reasoning). This evidence is especially unexpected concerning sustained attention, which was the cognitive function more consistently reported to benefit from short CPAP treatment (i.e., 1–15 days; [3,14,15]). In our study, we used the computerized Attention Network Test [38], which effectively evaluates three attentional networks simultaneously (alerting, orienting, and executive networks), providing separate, but interrelated, scores based on a precise recording of participants’ reaction times according to the patients’ behavior. This approach is hardly comparable with the traditional neuropsychological assessment of attention, as measured by pencil-and-paper tests, thus, possibly explaining the inconsistencies with previous findings. Then, we may encourage the use of computerized and easy-to-follow procedures to assess attention, instead using exclusively traditional pencil-and-paper tests. Because of the very simple instructions, the Attention Network Test [38] is an open-source instrument that can be easily used across different age groups, educational levels, and pathological conditions [52,53,54,55]. Nonetheless, normative standardized data about this test should be necessary for its adoption also in clinical contexts. Additionally, our results suggest that the short-term use of CPAP was not related to the cognitive speed of information processing in our sample, as measured by the Symbol Digit Modalities Test [37]; this evidence is in line with the results reported by Lim and colleagues (2007): the authors registered a benefit in cognitive functioning after two weeks of CPAP treatment. Overall, we may suggest that the possible dissimilar sensitivity of the measures adopted across different investigations may explain the inconsistencies found relative to previous studies, indicating that certain tests may be more suitable to detect short-term, subtle changes in cognitive functioning than others. Identifying those instruments more effective in detecting the possible impact of a (even short) treatment represents the first step to fruitful research in the field; hence, we suggest future studies should aim for this purpose.

It is interesting to note that that higher-order cognitive functions, such as strategic reasoning, problem-solving, mental planning (i.e., here measured by the Tower of London [39]) and the attentional executive control (here measured by the Attention Network Test [38]) were not related to a short CPAP treatment. Our results, which were in line with previous evidence [3,14,16], may suggest that higher-order cognitive domains either require longer treatment to improve or they cannot be restored. The oxidative stress related to sleep apneas severely and negatively affects the brain structures and functioning, through different pathological processes such as the consequent vasogenic edema, the alteration of the extracellular/intracellular fluid balance, and neurons loss [56]. The side-effect of nocturnal hypoxia on the brain has been reported in different and several cerebral areas [57], explaining the heterogeneity of cognitive deficits observed in OSAS. However, these areas may not be equally sensitive to the restoring of the nocturnal oxygen intake. Likely, several nights of normal breathing are needed for the edema reabsorption, explaining the benefit of a prolonged use of the CPAP. On the other hand, residual cognitive deficits after long-term and even multidisciplinary (i.e., ventilotherapy and cognitive rehabilitation) treatments may reflect irreversible anoxic damage to the CNS [5]. On the contrary, certain neural populations in the hippocampus, which is a key structure supporting memory and learning, are still capable of neurogenesis in adulthood, although this process seems inhibited by sleep fragmentation [57]. We speculate that effectively reducing hypoxia while sleeping and improving sleep continuity may promote neurogenesis in the hippocampus, but not in other brain areas, rapidly counteracting the consequences of hypoxia. Eventually, this may explain why only learning and verbal memory were significantly associated with the days of CPAP treatment in our study. Nonetheless, it is conceivable that factors other than reduced hypoxia, which are secondary to ventilotherapy, possibly contributed to the association between the treatment and cognitive performance. Valencia Flores and colleagues (1996) observed that improvements in cognition (i.e., in verbal learning) after CPAP ventilotherapy were the result of improved vigilance, not of the treatment per se. Since in our study, we did not specifically assess the level of vigilance, we cannot exclude that higher scores in verbal learning, as well as in the long-term memory, may be due to increased vigilance or energy because of improved sleep quality. Indeed, it should be clarified whether a few days of CPAP use might be enough to counteract the side effect of hypoxia on the brain. From a clinical perspective, we may suggest assessing multiple cognitive domains in the neuropsychological assessment of OSAS, as well as managing rehabilitative cognitive interventions in parallel with the CPAP treatment, specifically addressing these cognitive domains which might be less susceptible to changes because of the ventilotherapy.

Other final considerations can be made about our results. Age and the body mass index (which is informative of the level of obesity) were related to the cognitive functioning in our sample. As both age and body mass index increase, the efficiency of executive control decreases (i.e., reaction time associated with executive control increases, here measured by the Attention Network Test [38]); furthermore, aging was negatively related to the information processing speed (i.e., here measured by the Symbol Digit Modalities Test [37]), verbal learning, and long-term memory (i.e., here measured by the Selective Reminding Test [36]). This may not be surprising due to the documented detrimental role of aging and obesity on cognition [58,59,60,61,62]. Indeed, aging is associated with an increased risk of many debilitating conditions (i.e., vascular factors, atherosclerosis, stroke, and inflammatory processes) as well as neurologic and psychiatric diseases, which all possibly affect cognition. Nonetheless, milder cognitive difficulties are also expected as a mere consequence of senescence (i.e., normative aging) [63]. As for obesity, there is growing evidence that it is associated with structural changes in the brain, regardless of the presence of neurodegenerative diseases [64,65]. Conceivably, these anatomical alterations could cause the cognitive difficulties detected in patients suffering from obesity, mainly attributable to attention and executive function deficits, as well as to memory, decision-making, reasoning, and visuospatial difficulties [33], with important consequences on individuals’ quality of life [66] and eating behaviors (i.e., food-related impulsivity) [67]. To sum up, aging and high body mass index might blunt the possible effect of the CPAP treatment, interfering with the interpretation of its effectiveness in the research setting and requiring extra care when tailoring complementary neuropsychological interventions in the clinical context. The possible concurrent effect of these components, at least on certain cognitive functions, should be carefully taken into consideration by both researchers and clinicians, especially since older age and obesity are both risk factors for the development of OSAS.

Finally, the level of anxiety and depression measured in our study was not linked to cognitive performance, in contrast with some previous evidence suggesting that psychological functioning may impact cognition [41,42]. However, it should be considered that in our case these variables were studied as possible concurrent predictors in the regression model (i.e., together with demographic and clinical indices), not as key independent variables. Moreover, the levels of anxiety and depressive symptoms in our sample were, on average, below the clinical cut-off, suggesting that our participants may suffer from very low levels of psychological distress.

Concerning the study’s limitations, the retrospective design represents a major drawback of this investigation. We could not plan pre- (i.e., baseline) and post-treatment evaluations at certain time points for all participants; indeed, we could not manage the variability or stratification of the number of days of CPAP treatment at the moment of testing. Furthermore, the retrospective and observational design adopted does not allow causal inferences about the effect of the CPAP treatment on cognition. Nevertheless, our purpose was to preliminarily explore the possible contribution of short-term CPAP to clarify whether an association would be found, acknowledging which cognitive domains would be involved the most. This preliminary knowledge may guide the design of randomized controlled trials or longitudinal investigations including pre- and post-treatment evaluations of patients’ cognitive profiles at certain time points, in comparison with individuals who did not receive any CPAP treatment. Additionally, the analyses performed relative to sustained attention, the strategic reasoning, and mental planning, were relatively underpowered, possibly explaining the absence of any significant predictive contribution of the CPAP treatment, and of the other independent variables considered, on these cognitive domains. However, considering the retrospective design of this study we could not increase the sample size.

## 5. Conclusions

Our results seem to suggest that ventilotherapy in OSAS might be associated with short-term benefits on individuals’ cognitive functioning, especially about verbal learning, and long-term verbal memory. This may encourage both patients and clinicians to promptly start the treatment, as it may lead to a spontaneous reversibility of some cognitive difficulties, then target those impairments, which might be less affected by the CPAP therapy, with focused neuropsychological rehabilitative interventions. Indeed, more effective multidisciplinary protocols, whose short-term benefits can be clearly communicated to the patient, may improve individuals’ compliance and engagement, leading to better clinical outcomes.

## Figures and Tables

**Table 1:** Participants grouped for days of CPAP usage at the moment of the neuropsychological assessment. Days of CPAP treatment, number of participants, and percentages relative to the whole sample (*n* = 50) are reported.

Days of CPAP Treatment	*n* of Participants	%
0 days (no treatment)	5	10%
1–5 days	22	44%
6–10 days	18	36%
11–15 days	4	8%

**Table 2:** Means, standard deviations (SD), and range values of the neuropsychological and psychological tests’ scores in participants with OSAS (*n* = 50). *SRT-LTS:* Selective Reminding Test—Long-Term Storage; SRT-CLTR: Selective Reminding Test—Consistency of Long-Term Retrieval; SRT-D: Selective Reminding Test—Delay Recall; ANT-Al: Attention Network Test—Alert; ANT-Or: Attention Network Test—Orienting; ANT-Ex: Attention Network Test—Executive Control; SDMT: Symbol Digit Modality Test as Measure of Information Processing Speed; ToL-Time: Tower of London—Time; ToL-Acc: Tower of London—Accuracy as Measure of Strategic Reasoning, Problem-Solving, and Mental Planning. STAI-Y2: State-Trait Anxiety Inventory form Y2; BDI: Beck Depression Inventory.

Test	Mean	SD	Minimum	Maximum
SRT-LTS	34.86	15.52	8	64
SRT-CLTR	26.54	15.42	2	61
SRT-D	6.90	2.87	2	12
ANT-Al	−17.16	44.74	−119.54	69.75
ANT-Or	−33.29	40.50	−107.93	98.71
ANT-Ex	160.17	50.86	84.56	295.08
SDMT	46.10	11.49	23	78
ToL-Time	30.48	3.48	20	36
ToL-Acc	30.42	3.28	19	36
STAI-Y2	38.04	9.98	20	66
BDI	5.92	5.22	0	22

**Table 3:** Results of the hierarchical multiple linear regression analysis investigating the possible predictors of the long-term memory storage capacity (i.e., Selective Reminding Test—LTS score). Adjusted R^2^ and F-statistics are reported for each model. Standardized Beta, *t*-values, *p*-values, and partial *r* correlation coefficients are reported for each predictor in each model. BMI: body mass index; AHI: apnea-hypopnea index; % Time SpO_2_ < 90%: cumulative time spent with peripheral blood oxygen saturation (SpO_2_) below 90%; STAI-Y2: State-Trait Anxiety Inventory form Y2; BDI: Beck Depression Inventory. Bold means significant results (*p* ≤ 0.05).

		Beta	*t*-Value	*p*-Value	Partial *r*
**Model 1**	Adjust R^2^ = 0.32 (F_6,43_ = 4.91, *p* = 0.001)				
	(Constant)		1.77	0.083	
	Sex	0.10	0.79	0.435	0.12
	Age	−0.40	−2.80	**0.008**	−0.39
	Education	0.26	1.85	0.072	0.27
	BMI	0.13	1.01	0.318	0.15
	AHI	0.18	1.29	0.204	0.19
	% Time SpO_2_ < 90%	−0.02	−0.17	0.862	−0.03
**Model 2**	Adjust R^2^ = 0.30 (F_8,41_ = 3.59, *p* = 0.003)				
	(Constant)		1.67	0.103	
	Sex	0.10	0.78	0.440	0.12
	Age	−0.39	−2.66	**0.011**	−0.38
	Education	0.26	1.82	0.077	0.27
	BMI	0.14	1.01	0.317	0.16
	AHI	0.19	1.31	0.199	0.20
	% Time SpO_2_ < 90%	−0.03	−0.24	0.810	−0.04
	STAI-Y2	0.01	0.07	0.943	0.01
	BDI	−0.08	−0.52	0.607	−0.08
**Model 3**	Adjust R^2^ = 0.35 (F_9,40_ = 3.98, *p* = 0.001)				
	(Constant)		2.41	0.021	
	Sex	0.11	0.88	0.382	0.14
	Age	−0.48	−3.23	**0.002**	−0.46
	Education	0.14	0.91	0.369	0.14
	BMI	0.02	0.12	0.908	0.02
	AHI	0.27	1.87	0.069	0.28
	% Time SpO_2_ < 90%	−0.03	−0.21	0.833	−0.03
	STAI-Y2	−0.09	−0.57	0.572	−0.09
	BDI	0.03	0.19	0.852	0.03
	Days of Adaptation	0.31	2.14	**0.039**	0.32

**Table 4:** Results of the hierarchical multiple linear regression analysis investigating the possible predictors of the consistency of long-term memory retrieval (i.e., Selective Reminding Test—CLTR score). Adjusted R^2^ and F-statistics are reported for each model. Standardized Beta, *t*-values, *p*-values, and partial *r* correlation coefficients are reported for each predictor in each model. BMI: body mass index; AHI: apnea-hypopnea index; % Time SpO_2_ < 90%: cumulative time spent with peripheral blood oxygen saturation (SpO_2_) below 90%; STAI-Y2: State-Trait Anxiety Inventory form Y2; BDI: Beck Depression Inventory. Bold means significant results (*p* ≤ 0.05).

		Beta	*t*-Value	*p*-Value	Partial *r*
**Model 1**	Adjust R^2^ = 0.39 (F_6,43_ = 6.26, *p* = 0.001)				
	(Constant)		1.67	0.103	
	Sex	0.09	0.71	0.481	0.11
	Age	−0.42	−3.10	**0.003**	−0.43
	Education	0.36	2.71	**0.010**	0.38
	BMI	0.09	0.71	0.481	0.11
	AHI	0.10	0.71	0.479	0.11
	% Time SpO_2_ < 90%	−0.02	−0.19	0.850	−0.03
**Model 2**	Adjust R^2^ = 0.39 (F_8,41_ = 4.94, *p* = 0.001)				
	(Constant)		1.47	0.150	
	Sex	0.08	0.66	0.516	0.10
	Age	−0.40	−2.93	**0.005**	−0.42
	Education	0.36	2.70	**0.010**	0.39
	BMI	0.09	0.71	0.481	0.11
	AHI	0.11	0.84	0.404	0.13
	% Time SpO_2_ < 90%	−0.05	−0.37	0.716	−0.06
	STAI-Y2	0.09	0.60	0.550	0.09
	BDI	−0.20	−1.38	0.175	−0.21
**Model 3**	Adjust R^2^ = 0.44 (F_9,40_ = 5.20, *p* = 0.001)				
	(Constant)		2.18	0.035	
	Sex	0.09	0.75	0.458	0.12
	Age	−0.48	−3.47	**0.001**	−0.48
	Education	0.25	1.78	0.082	0.27
	BMI	−0.02	−0.14	0.891	−0.02
	AHI	0.19	1.37	0.177	0.21
	% Time SpO_2_ < 90%	−0.04	−0.34	0.735	−0.05
	STAI-Y2	0.00	−0.02	0.987	0.00
	BDI	−0.10	−0.68	0.497	−0.11
	Days of Adaptation	0.27	2.05	**0.047**	0.31

**Table 5:** Results of the hierarchical multiple linear regression analysis investigating the possible predictors of the delay recall ability (i.e., Selective Reminding Test—D score). Adjusted R^2^ and F-statistics are reported for each model. Standardized Beta, *t*-values, *p*-values, and partial *r* correlation coefficients are reported for each predictor in each model. BMI: body mass index; AHI: apnea-hypopnea index; % Time SpO_2_ < 90%: cumulative time spent with peripheral blood oxygen saturation (SpO_2_) below 90%; STAI-Y2: State-Trait Anxiety Inventory form Y2; BDI: Beck Depression Inventory. Bold means significant results (*p* ≤ 0.05).

		Beta	*t*-Value	*p*-Value	Partial *r*
**Model 1**	Adjust R^2^ = 0.37 (F_6,43_ = 5.70, *p* = 0.001)				
	(Constant)		2.04	**0.047**	
	Sex	0.15	1.25	0.219	0.19
	Age	−0.38	−2.71	**0.010**	−0.38
	Education	0.41	2.99	**0.005**	0.41
	BMI	0.10	0.78	0.437	0.12
	AHI	−0.02	−0.18	0.860	−0.03
	% Time SpO_2_ < 90%	−0.12	−0.95	0.346	−0.14
**Model 2**	Adjust R^2^ = 0.34 (F_8,41_ = 4.13, *p* = 0.001)				
	(Constant)		1.83	0.074	
	Sex	0.14	1.10	0.277	0.17
	Age	−0.38	−2.62	**0.012**	−0.38
	Education	0.41	2.90	**0.006**	0.41
	BMI	0.09	0.70	0.490	0.11
	AHI	−0.02	−0.13	0.896	−0.02
	% Time SpO_2_ < 90%	−0.12	−0.96	0.342	−0.15
	STAI-Y2	0.07	0.48	0.636	0.07
	BDI	−0.06	−0.38	0.705	−0.06
**Model 3**	Adjust R^2^ = 0.40 (F_9,40_ = 4.60, *p* = 0.001)				
	(Constant)		2.62	**0.012**	
	Sex	0.15	1.23	0.226	0.19
	Age	−0.46	−3.22	**0.003**	−0.45
	Education	0.28	1.92	**0.062**	0.29
	BMI	−0.03	−0.22	0.824	−0.04
	AHI	0.06	0.45	0.658	0.07
	% Time SpO_2_ < 90%	−0.12	−0.96	0.340	−0.15
	STAI-Y2	−0.03	−0.19	0.847	−0.03
	BDI	0.05	0.35	0.725	0.06
	Days of Adaptation	0.31	3.23	**0.031**	0.33

**Table 6:** Results of the hierarchical multiple linear regression analysis investigating the possible predictors of processing speed (i.e., SDMT score). Adjusted R^2^ and F-statistics are reported for each model. Standardized Beta, *t*-values, *p*-values, and partial *r* correlation coefficients are reported for each predictor in each model. BMI: body mass index; AHI: apnea-hypopnea index; % Time SpO_2_ < 90%: cumulative time spent with peripheral blood oxygen saturation (SpO_2_) below 90%; STAI-Y2: State-Trait Anxiety Inventory form Y2; BDI: Beck Depression Inventory. Bold means significant results (*p* ≤ 0.05).

		Beta	*t*-Value	*p*-Value	Partial *r*
**Model 1**	Adjust R2 = 0.30 (F_6,43_ = 4.47, *p* = 0.001)				
	(Constant)		4.19	0.000	
	Sex	0.06	0.47	0.640	0.07
	Age	−0.45	−3.07	**0.004**	−0.42
	Education	0.21	1.46	0.153	0.22
	BMI	−0.09	−0.68	0.501	−0.10
	AHI	0.18	1.26	0.214	0.19
	% Time SpO_2_ < 90%	−0.04	−0.27	0.791	−0.04
**Model 2**	Adjust R2 = 0.27 (F_8,41_ = 3.23, *p* = 0.006)				
	(Constant)		3.92	0.000	
	Sex	0.06	0.41	0.682	0.06
	Age	−0.45	−2.96	**0.005**	−0.42
	Education	0.21	1.41	0.166	0.22
	BMI	−0.09	−0.67	0.505	−0.10
	AHI	0.19	1.27	0.213	0.19
	% Time SpO_2_ < 90%	−0.04	−0.30	0.764	−0.05
	STAI-Y2	0.04	0.27	0.791	0.04
	BDI	−0.06	−0.37	0.710	−0.06
**Model 3**	Adjust R2 = 0.27 (F_9,40_ = 3.12, *p* = 0.006)				
	(Constant)		3.17	0.003	
	Sex	0.05	0.37	0.712	0.06
	Age	−0.39	−2.54	**0.015**	−0.37
	Education	0.29	1.83	0.075	0.28
	BMI	−0.01	−0.10	0.924	−0.02
	AHI	0.14	0.89	0.378	0.14
	% Time SpO_2_ < 90%	−0.04	−0.33	0.743	−0.05
	STAI-Y2	0.11	0.65	0.518	0.10
	BDI	−0.13	−0.79	0.435	−0.12
	Days of Adaptation	−0.20	−1.32	0.194	−0.20

**Table 7:** Results of the hierarchical multiple linear regression analysis investigating the possible predictors of the efficiency of the executive attention network (i.e., ANT-Ex score *). Adjusted R2 and F-statistics are reported for each model. Standardized Beta, t-values, *p*-values, and partial r correlation coefficients are reported for each predictor in each model. BMI: body mass index; AHI: apnea-hypopnea index; % Time SpO_2_ < 90%: cumulative time spent with peripheral blood oxygen saturation (SpO_2_) below 90%; STAI-Y2: State-Trait Anxiety Inventory form Y2; BDI: Beck Depression Inventory. * refers to the results relative to to the logarithmic transformation of *ANT- Ex* scores. Bold means significant results (*p* ≤ 0.05).

		Beta	*t*-Value	*p*-Value	Partial *r*
**Model 1**	Adjust R^2^ = 0.20 (F_6,43_ = 3.10, *p* = 0.01)				
	(Constant)		7.57	0.000	
	Sex	0.12	0.86	0.393	0.13
	Age	0.45	2.87	**0.006**	0.40
	Education	0.03	0.18	0.858	0.03
	BMI	0.29	2.03	**0.049**	0.30
	AHI	−0.05	−0.31	0.756	−0.05
	% Time SpO_2_ < 90%	0.14	1.02	0.313	0.15
**Model 2**	Adjust R^2^ = 0.21 (F_8,41_ = 2.60, *p* = 0.02)				
	(Constant)		7.20	0.000	
	Sex	0.08	0.60	0.552	0.09
	Age	0.43	2.74	**0.009**	0.39
	Education	0.02	0.11	0.912	0.02
	BMI	0.26	1.79	0.080	0.27
	AHI	−0.05	−0.35	0.731	−0.05
	% Time SpO_2_ < 90%	0.16	1.12	0.269	0.17
	STAI-Y2	0.13	0.78	0.440	0.12
	BDI	0.08	0.50	0.617	0.08
**Model 3**	Adjust R^2^ = 0.25 (F_9,40_ = 2.81, *p* = 0.01)				
	(Constant)		6.19	0.000	
	Sex	0.08	0.55	0.583	0.09
	Age	0.51	3.19	**0.003**	0.45
	Education	0.13	0.82	0.418	0.13
	BMI	0.37	2.42	**0.020**	0.36
	AHI	−0.13	−0.82	0.419	−0.13
	% Time SpO_2_ < 90%	0.15	1.12	0.271	0.17
	STAI-Y2	0.22	1.31	0.197	0.20
	BDI	−0.02	−0.11	0.915	−0.02
	Days of Adaptation	−0.28	−1.83	0.075	−0.28

## Data Availability

The dataset generated and analyzed during the current study is available in the Zenodo repository (10.5281/zenodo.7372109) on reasonable request.

## Supplementary Materials

The following supporting information can be downloaded at: https://www.mdpi.com/article/10.3390/brainsci13010124/s1, Table S1: Results of the hierarchical multiple linear regression analysis investigating the possible predictors of the efficiency of the alerting attention network (i.e., the *Attention Network Test—Alerting* score); Table S2: Results of the hierarchical multiple linear regression analysis investigating the possible predictors of the efficiency of the orienting attention network (i.e., the *Attention Network Test—Orienting* score); Table S3: Results of the hierarchical multiple linear regression analysis investigating the possible predictors of the speed associated with strategic reasoning, problem-solving, and mental planning (i.e., *Tower of London—Time* score); Table S4: Results of the hierarchical multiple linear regression analysis investigating the possible predictors of the accuracy of strategic reasoning, problem-solving, and mental planning (i.e., Tower of London—Accuracy score).
