# Supplementary material for: Short-Term Benefits of Continuous Positive Airway Pressure Treatment on Cognition in the Obstructive Sleep Apnea Syndrome: A Retrospective Study
Source: Brain Sci. 2023 Jan 10;13(1):124. doi: 10.3390/brainsci13010124 (PMC9856474; doi:10.3390/brainsci13010124)
Supplement: Supplementary file 1 [file brainsci-13-00124-s001.zip › brainsci-2094341-supplementary.pdf]

Short-term benefits of continuous positive airway pressure treatment on cognition in the obstructive sleep apnea syndrome: a retrospective study.

Table S1. Results of the hierarchical multiple linear regression analysis investigating the possible predictors of the efficiency of the alerting attention network (i.e., the *Attention Network Test - Alerting* score). Adjust R^2 and F-statistics are reported for each model. Standardized Beta, *t*-values, *p*-values and partial *r* correlation coefficients are reported for each predictor in each model. *BMI:* body mass index; *AHI*: apnea-hypopnea index; *% Time SpO2 < 90%*: cumulative time spent with peripheral blood oxygen saturation (SpO2) below 90%; *STAI-Y2:* State-Trait Anxiety Inventory form Y2; *BDI:* Beck Depression Inventory.

		**Beta**	***t*-value**	***p*-value**	**Partial *r***
**Model 1**	Adjust R^2 = 0.03 ($F_{6,43}$ =1.25, p = 0.30)				
	(Constant)		1.07	0.290	
	Sex	-0.19	-1.27	0.211	-0.19
	Age	0.03	0.17	0.868	0.03
	Education	-0.08	-0.45	0.652	-0.07
	BMI	-0.30	-1.89	0.066	-0.28
	AHI	-0.09	-0.55	0.588	-0.08
	% Time SpO2 < 90%	-0.03	-0.21	0.838	-0.03
**Model 2**	Adjust R^2 = 0.05 ($F_{8,41}$ =1.31, p =0.26)				
	(Constant)		0.73	0.471	
	Sex	-0.22	-1.46	0.152	-0.22
	Age	0.05	0.29	0.770	0.05
	Education	-0.09	-0.51	0.611	-0.08
	BMI	-0.32	-2.00	0.053	-0.30
	AHI	-0.07	-0.38	0.702	-0.06
	% Time SpO2 < 90%	-0.06	-0.39	0.700	-0.06
	STAI-Y2	0.25	1.34	0.187	0.21
	BDI	-0.29	-1.61	0.110	-0.24
**Model 3**	Adjust R^2 = 0.03 ($F_{9,40}$ =1.14, p =0.36)				
	(Constant)		0.63	0.532	
	Sex	-0.22	-1.45	0.156	-0.22
	Age	0.06	0.31	0.760	0.05
	Education	-0.08	-0.43	0.671	-0.07

BMI	-0.31	-1.77	0.085	-0.27
AHI	-0.07	-0.39	0.696	-0.06
% Time SpO2 < 90%	-0.06	-0.39	0.702	-0.06
STAI-Y2	0.25	1.30	0.203	0.20
BDI	-0.30	-1.54	0.131	-0.24
Days of Adaptation	-0.02	-0.10	0.921	-0.02

Table S2. Results of the hierarchical multiple linear regression analysis investigating the possible predictors of the efficiency of the orienting attention network (i.e., the *Attention Network Test - Orienting* score). Adjust R^2 and F-statistics are reported for each model. Standardized Beta, *t*-values, *p*-values and partial *r* correlation coefficients are reported for each predictor in each model. *BMI:* body mass index; *AHI*: apnea-hypopnea index; *% Time SpO2 < 90%*: cumulative time spent with peripheral blood oxygen saturation (SpO2) below 90%; *STAI-Y2:* State-Trait Anxiety Inventory form Y2; *BDI:* Beck Depression Inventory.

		Beta	*t*-value	*p*-value	Partial *r*
**Model 1**	Adjust $R^2 = 0.16$ ($F^{6,43} = 2.52$, $p = 0.04$)				
	(Constant)		0.96	0.343	
	Sex	-0.09	-0.62	0.538	-0.09
	Age	0.13	0.82	0.415	0.12
	Education	-0.36	-2.30	**0.026**	-0.33
	BMI	-0.34	-2.30	**0.026**	-0.33
	AHI	0.09	0.59	0.557	0.09
	% Time SpO2 < 90%	-0.08	-0.54	0.595	-0.08
**Model 2**	Adjust $R^2 = 0.13$ ($F_{8,41} = 1.95$, $p = 0.08$)				
	(Constant)		0.96	0.343	
	Sex	-0.09	-0.64	0.529	-0.10
	Age	0.12	0.71	0.483	0.11
	Education	-0.37	-2.28	**0.028**	-0.34
	BMI	-0.35	-2.29	**0.027**	-0.34
	AHI	0.08	0.51	0.615	0.08
	% Time SpO2 < 90%	-0.06	-0.41	0.684	-0.06
	STAI-Y2	-0.02	-0.12	0.903	-0.02
	BDI	0.14	0.82	0.419	0.13

**Model 3**	Adjust R^2 = 0.17 ($F_{9,40}$ = 2.12, p = 0.051)				
	(Constant)		1.53	0.134	
	Sex	-0.09	-0.59	0.557	-0.09
	Age	0.05	0.27	0.787	0.04
	Education	-0.48	-2.79	0.008	-0.40
	BMI	-0.46	-2.81	0.008	-0.41
	AHI	0.15	0.93	0.358	0.15
	% Time SpO2 < 90%	-0.06	-0.39	0.701	-0.06
	STAI-Y2	-0.11	-0.62	0.539	-0.10
	BDI	0.24	1.33	0.190	0.21
	Days of Adaptation	0.27	1.67	0.104	0.25

Table S3. Results of the hierarchical multiple linear regression analysis investigating the possible predictors of the speed associated with strategical reasoning, problem-solving, and mental planning (i.e., *Tower of London -Time* score*). Adjust R^2 and F-statistics are reported for each model. Standardized Beta, *t*-values, *p*-values and partial *r* correlation coefficients are reported for each predictor in each model. *BMI:* body mass index; *AHI*: apnea-hypopnea index; *% Time SpO2 < 90%*: cumulative time spent with peripheral blood oxygen saturation (SpO2) below 90%; *STAI-Y2:* State-Trait Anxiety Inventory form Y2; *BDI:* Beck Depression Inventory. Results refer to the logarithmic transformation of *Tower of London – Time score.*

		**Beta**	***t*-value**	***p*-value**	**Partial *r***
**Model 1**	Adjust R^2 =0.01 ($F_{6,43}$ =1.04, p = 0.41)				
	(Constant)		16.83	0.000	
	Sex	0.12	0.78	0.440	0.12
	Age	-0.29	-1.68	0.101	-0.25
	Education	-0.05	-0.27	0.790	-0.04
	BMI	0.06	0.39	0.698	0.06
	AHI	0.06	0.38	0.707	0.06
	% Time SpO2 < 90%	0.20	1.26	0.214	0.19
**Model 2**	Adjust R^2 =0.003 ($F_{8,41}$ =1.02, p = 0.44)				
	(Constant)		16.10	0.000	

	Sex	0.07	0.48	0.635	0.07
	Age	-0.28	-1.61	0.115	-0.24
	Education	-0.06	-0.35	0.732	-0.05
	BMI	0.03	0.20	0.845	0.03
	AHI	0.09	0.51	0.612	0.08
	% Time SpO2 < 90%	0.18	1.14	0.262	0.17
	STAI-Y2	0.28	1.52	0.137	0.23
	BDI	-0.22	-1.24	0.221	-0.19
**Model 3**	Adjust R^2 =0.04 ($F_{9,40}$ =1.22, p = 0.31)				
	(Constant)		14.62	0.000	
	Sex	0.07	0.43	0.667	0.07
	Age	-0.21	-1.18	0.247	-0.18
	Education	0.05	0.30	0.763	0.05
	BMI	0.14	0.83	0.414	0.13
	AHI	0.02	0.09	0.931	0.01
	% Time SpO2 < 90%	0.17	1.13	0.266	0.18
	STAI-Y2	0.37	1.96	0.057	0.30
	BDI	-0.32	-1.72	0.093	-0.26
	Days of Adaptation	-0.28	-1.61	0.115	-0.25

Table S4. Results of the hierarchical multiple linear regression analysis investigating the possible predictors of the accuracy of strategical reasoning, problem-solving, and mental planning (i.e., *Tower of London - Accuracy* score). Adjust R^2 and F-statistics are reported for each model. Standardized Beta, *t*-values, *p*-values and partial *r* correlation coefficients are reported for each predictor in each model. *BMI:* body mass index; *AHI*: apnea-hypopnea index; *% Time SpO2 < 90%*: cumulative time spent with peripheral blood oxygen saturation (SpO2) below 90%; *STAI-Y2:* State-Trait Anxiety Inventory form Y2; *BDI:* Beck Depression Inventory.

		**Beta**	***t*-value**	***p*-value**	**Partial *r***
**Model 1**	Adjust R^2 =0.04 ($F_{6,43}$ =1.31, p =0.28)				
	(Constant)		4.79	0.000	
	Sex	0.03	0.21	0.833	0.03

	Age	-0.16	-0.95	0.347	-0.14
	Education	0.11	0.67	0.505	0.10
	BMI	0.22	1.37	0.176	0.21
	AHI	0.00	-0.01	0.995	0.00
	% Time SpO2 < 90%	0.21	1.35	0.184	0.20
**Model 2**	Adjust R^2 =0.09 ($F_{8,41}$ =1.64, p =0.15)				
	(Constant)		4.31	0.000	
	Sex	-0.03	-0.19	0.853	-0.03
	Age	-0.15	-0.89	0.381	-0.14
	Education	0.10	0.59	0.561	0.09
	BMI	0.18	1.13	0.265	0.17
	AHI	0.03	0.18	0.861	0.03
	% Time SpO2 < 90%	0.19	1.22	0.230	0.19
	STAI-Y2	0.38	2.11	0.041	0.31
	BDI	-0.30	-1.71	0.095	-0.26
**Model 3**	Adjust R^2 =0.12 ($F_{9,40}$ =1.71, p =0.12)				
	(Constant)		3.52	0.001	
	Sex	-0.03	-0.24	0.815	-0.04
	Age	-0.09	-0.50	0.616	-0.08
	Education	0.19	1.10	0.279	0.17
	BMI	0.27	1.61	0.115	0.25
	AHI	-0.03	-0.19	0.848	-0.03
	% Time SpO2 < 90%	0.18	1.21	0.235	0.19
	STAI-Y2	0.46	2.46	0.018	0.36
	BDI	-0.38	-2.10	0.042	-0.31
	Days of Adaptation	-0.24	-1.41	0.165	-0.22
